# Sustainable Skincare Innovation: Cork Powder Extracts as Active Ingredients for Skin Aging

**DOI:** 10.3390/ph18010121

**Published:** 2025-01-17

**Authors:** Ana Silva, Cláudia Pinto, Sara Cravo, Sandra Mota, Liliana Rego, Smeera Ratanji, Clara Quintas, Joana Rocha e Silva, Carlos Afonso, Maria Elizabeth Tiritan, Honorina Cidade, Teresa Cruz, Isabel F. Almeida

**Affiliations:** 1 Faculty of Pharmacy, University of Coimbra, 3000-548 Coimbra, Portugal; anacrs@cnc.uc.pt; 2 CIBB—Center for Innovative Biomedicine and Biotechnology/CNC—Center for Neuroscience and Cell Biology, University of Coimbra, 3004-504 Coimbra, Portugal; 3 Laboratory of Organic and Pharmaceutical Chemistry, Department of Chemical Sciences, Faculty of Pharmacy, University of Porto, Rua Jorge Viterbo Ferreira 228, 4050-313 Porto, Portugal; up201604041@ff.up.pt (C.P.); scravo@ff.up.pt (S.C.); cafonso@ff.up.pt (C.A.); beth@ff.up.pt (M.E.T.); 4 CIIMAR–Interdisciplinary Centre of Marine and Environmental Research, University of Porto, Avenida General Norton de Matos, S/N, 4450-208 Matosinhos, Portugal; 5 UCIBIO—Applied Molecular Biosciences Unit, Department of Drug Sciences, Faculty of Pharmacy, University of Porto, R. Jorge Viterbo Ferreira 228, 4050-313 Porto, Portugal; up201608486@edu.ff.up.pt (S.M.); up201203377@edu.ff.up.pt (L.R.); ifalmeida@ff.up.pt (I.F.A.); 6 Associate Laboratory i4HB—Institute for Health and Bioeconomy, Faculty of Pharmacy, University of Porto, R. Jorge Viterbo Ferreira 228, 4050-313 Porto, Portugal; up201805318@edu.ff.up.pt (S.R.); claraquintas@ff.up.pt (C.Q.); 7 UCIBIO—Applied Molecular Biosciences Unit, Laboratory of Pharmacology, Department of Drug Sciences, Faculty of Pharmacy, University of Porto, R. Jorge Viterbo Ferreira 228, 4050-313 Porto, Portugal; 8 Dimas & Silva, Lda. Industry, Rua Central de Goda 345, 4535-167 Mozelos, Portugal; joanasilva@dimas-silva.pt; 9 TOXRUN—Toxicology Research Unit, University Institute of Health Sciences, CESPU, CRL, 4585-116 Gandra, Portugal; 10 UNIPRO—Oral Pathology and Rehabilitation Research Unit, University Institute of Health Sciences (CESPU), 4585-116 Gandra, Portugal

**Keywords:** cosmetics, cork extracts, skin aging, sustainability, senescence, inflammaging, circular economy

## Abstract

**Background**: An emerging practice within the concept of circular beauty involves the upcycling of agro-industrial by-products. Cork processing, for instance, yields by-products like cork powder, which presents an opportunity to create value-added cosmetic ingredients. Building upon our previous research, demonstrating the antioxidant potential of hydroalcoholic extracts derived from two distinct cork powders (P0 and P1), in this work, aqueous extracts were prepared and analyzed. The safety and bioactivities of the newly obtained aqueous extracts, as well as the 30% ethanol extracts, previously reported to be the most promising for skin application, were also evaluated. **Methods**: Aqueous extracts were obtained from cork powders (P0 and P1) and the identification and quantification of some polyphenols was achieved by liquid chromatography (LC). Antioxidant potential was screened by DPPH method and the bioactivity and safety of extracts were further explored using cell-based assays. **Results**: All extracts exhibited a reduction in age-related markers, including senescence-associated beta-galactosidase (SA-β-gal) activity. Additionally, they demonstrated a pronounced anti-inflammatory effect by suppressing the production of several pro-inflammatory mediators in macrophages upon lipopolysaccharide stimulation. Moreover, the extracts upregulated genes and proteins associated with antioxidant activity, such as heme oxygenase 1. The aqueous extract from P1 powder was especially active in reducing pro-inflammatory mediators, namely the *Nos2* gene, inducible nitric oxide protein levels, and nitric oxide production. Moreover, it did not induce skin irritation, as assessed by the EpiSkin test, in compliance with the OECD Test Guidelines. **Conclusions**: Overall, our findings underscore the potential of aqueous extracts derived from cork waste streams to mitigate various hallmarks of skin aging, including senescence and inflammaging, and their suitability for incorporation into cosmetics formulations. These results warrant further exploration for their application in the pharmaceutical and cosmetic industries and could foster a sustainable and circular bioeconomy.

## 1. Introduction

The increase in life expectancy has heightened the desire to maintain a healthy lifestyle, driving the search for effective strategies to combat aging, particularly concerning skin health. Over time, the skin’s functions become compromised, leading to increased fragility, reduced elasticity, and diminished tensile strength. This decline results from the degradation of collagen and elastic fibers in the dermis, along with the disruption of cell–cell junctions in the epidermis [1]. The loss of responsiveness by the immune system, named immunosenescence, is accompanied by inflammatory events derived from the increased levels of inflammatory cytokines [2]. Nitric oxide (NO) is a signaling molecule with key biological roles, including vasodilation and neurotransmission, as well as crucial functions in innate immunity and inflammation [3]. The overexpression of the enzyme inducible nitric oxide synthase (iNOS), triggered by cytokines and pathogens, plays a central role in various inflammatory processes affecting multiple skin cell types. This overactivity contributes to the development of skin diseases such as psoriasis, cutaneous lupus erythematosus, systemic sclerosis, and dermatitis [3,4,5]. Thus, the search for natural products capable of exerting an inhibitory effect on iNOS expression through the modulation of transcription factors has become especially relevant to attenuating the effects caused by inflammation [6].

Sustainability is an increasingly imperative goal of cosmetics companies, partly due to consumers’ awareness of environmental issues, which drives demand for more sustainable products and practices. Consequently, the circular economy, defined by the European Parliament as “a model of production and consumption, which involves sharing, leasing, reusing, repairing, refurbishing and recycling existing materials and products as long as possible”, is fundamentally based on better management of natural resources, reducing the consumption of synthetic materials, and encouraging reuse and recycling, leading to an extended life cycle [7]. This issue has gained even more prominence with the 17 Sustainable Development Goals proposed by the United Nations to reach global prosperity by 2030. To become economically viable, the valorization of agri-food waste can be achieved through the production of useful and more eco-friendly bioactive ingredients for different industrial sectors, including cosmetic products [8,9].

The outer bark of *Quercus suber*, known as cork, is an important sustainable raw resource with numerous applications, including the production of cork stoppers, agglomerated cork, insulation, and flooring [10,11,12]. Nonetheless, cork manufacturing and processing generate a substantial amount of waste, presenting a significant challenge within the cork industry that requires effective solutions. Cork powder is regarded as the most important by-product of the industrial processing of cork oak bark after it has undergone virtually all transformations.

Several extracts of *Quercus* sp. have been reported for their potential biological activities, namely antioxidant and anti-inflammatory, among others [13,14,15]. Moreover, several studies have highlighted cork powder as a source of bioactive compounds from different classes, including phenolics, mainly with antioxidant and anti-inflammatory activities, and is of interest in the cosmetic industry for putative application in skincare products with anti-aging effects [16,17,18].

Considering the potential of cork powder to obtain extracts with promising biological properties for the cosmetic industry, and in the context of the circular economy, recently, our research team explored the antioxidant potential of hydroalcoholic extracts obtained from two distinct cork powders, coarse powder (P0) and fine powder (P1) [19]. For this purpose, three extracts were prepared with different percentages of ethanol (30 to 96%). The aim was to find a relationship between the presence of certain compounds and the antioxidant activity of the extract. We found that the 30% ethanol extracts of P0 and P1 demonstrated the highest α,α-diphenyl-β-picrylhydrazyl (DPPH) free radical scavenging activity (P0: IC_50_ = 7.0 ± 0.1 μg/mL, P1: IC_50_ = 4.7 ± 0.2 μg/mL), possibly attributed to their higher concentration of phenolic compounds, particularly vescalagin and castalagin. Building on the promising results of the 30% ethanol extracts, this study explores various skin-related bioactivities, aiming to enhance their value as upcycled products with potential applications in the cosmetic industry. Considering that the use of water represents a more sustainable and eco-friendly process, water extracts of P0 and P1 were also prepared and tested. The optimization of the extraction process took into account parameters such as temperature, extraction duration, and the number of extraction cycles, assessing their impact on both DPPH-scavenging activity and the overall sustainability of the process. Additional studies were carried out using liquid chromatography coupled with high-resolution mass spectrometry (LC-HRMS) and ultraviolet–visible (LC-UV) detection to identify and quantify specific compounds in the P0 and P1 aqueous extracts. The results were similar to those from 30% ethanol extracts. Finally, the skin anti-aging effects of all extracts were evaluated and preformulation studies were performed to expand the investigation of cork powder as a sustainable raw material for developing cosmetic ingredients.

## 2. Results

### 2.1. Preparation of Aqueous Cork Powder Extracts with DPPH-Scavenging Effect

**Figure 1:**
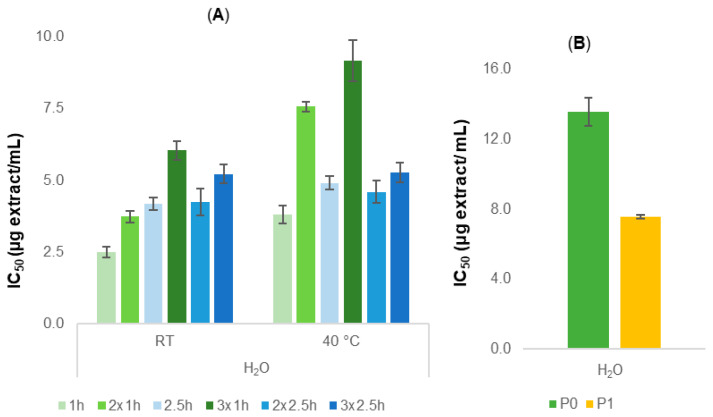
(**A**) IC_50_ values of P1 aqueous extracts expressed in μg/mL (mean ± SEM from 3 independent experiments). Ascorbic acid (positive control): IC_50_ = 7.9 ± 0.4 μg/mL. (**B**) IC_50_ values of P0 and P1 aqueous extracts expressed in μg/mL (mean ± SEM from 3 independent experiments).

The first step for the preparation of aqueous extracts consisted of the selection of extraction conditions to obtain sustainable extracts with potential anti-aging effects. Optimization of the extraction process considered the influence of parameters such as temperature, the duration of the extraction, and the number of extraction cycles on both DPPH-scavenging activity and the sustainability of the process. For this purpose, a sample of P1 cork powder was stirred at room temperature or 40 °C, using two different conditions, cycles of 1 h and cycles of 2.5 h with single and multiple extraction, and the DPPH-scavenging activity was tested (Figure 1A).

For all conditions, promising DPPH-scavenging activity was observed (IC_50_ < 9.1 ± 0.7 μg/mL), with the best scavenging activity obtained from the extract prepared at room temperature for 1 h (IC_50_ = 2.5 ± 0.2 μg/mL). Moreover, extracts prepared at room temperature with multiple extractions were associated with a reduction in the DPPH-scavenging effect, particularly from the second to the third extraction, as reported previously with the hydroalcoholic extracts [19]. To ensure sustainability and considering the results previously obtained, a one-hour extraction cycle at room temperature was used for future studies. After this, extracts obtained from both P0 and P1 powders were prepared at room temperature with 1 h of stirring using double the amount of water to improve the yield of extraction, which increased from 6% to 10%. The obtained extracts were once again assessed for their DPPH-scavenging effect. Both P0 and P1 aqueous extracts displayed promising DPPH-scavenging activity, similar to ascorbic acid used as a positive control (IC_50_ = 7.9 ± 0.4 μg/mL), with P1 extract (IC_50_ = 7.5 ± 0.1 μg/mL) being more active than P0 (IC_50_ = 13.5 ± 0.8 μg/mL) (Figure 1B).

### 2.2. Analysis of Extracts via LC

The cork powder extract composition was determined by a validated method using LC-UV. The identity of the target compounds was unequivocally confirmed by LC-HRMS. The exact mass data of the quantified compounds in these extracts are presented in Tables S1 and S2. The LC-UV analytical method was based on a recent publication [19], which also focuses on the sustainability of the analysis, considering the use of non-toxic solvents in the mobile phase as well as their reduction in consumption.

**Figure 2:**
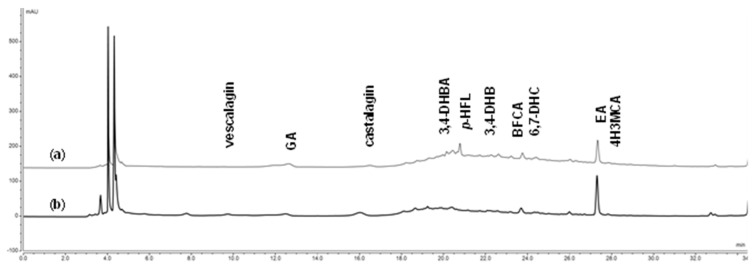
Compounds identified in P0 (**a**) and P1 (**b**) aqueous cork powder extracts. Vescalagin; gallic acid: GA; castalagin; protocatechuic acid: 3,4-DHBA; latifolicinin C acid: *p*-HFL; protocatechuic aldehyde: 3,4-DHB; brevifolincarboxylic acid: BFCA; aesculetin: 6,7-DHC; ellagic acid: EA; coniferyl aldehyde: 4H3MCA.

A chromatographic analysis of aqueous cork extracts showed the complexity of these extracts, as already shown in 30% ethanol extracts [19]. In these extracts, it was also possible to identify ten compounds belonging to the phenolic family, including vescalagin, gallic acid (GA), castalagin, protocatechuic acid (3,4-DHBA), latifolicinin C acid (*p*-HFL), protocatechuic aldehyde (3,4-DHB), brevifolincarboxylic acid (BFCA), aesculetin (6,7-DHC), ellagic acid (EA), and coniferyl aldehyde (4H3MCA) (Figure 2). However, only eight compounds could be quantified, and vescalagin and 4H3MCA were below their limit of quantification (LOQ) (Table 1). 

Compared to the P1 extract, the P0 extract showed more phenolic content and was therefore richer in phenolic acids, phenolic aldehydes, and coumarins. In this extract, the major compounds identified were the phenolic acids *p*-HFL and GA. Among all the phenolic acids, ellagic acid was the only one that showed lower quantities in the P0 extract than in the P1 extract.

On the other hand, P1 extract presented a higher content of tannins, with castalagin being the predominant compound. Vescalagin was identified on the P1 extract even though it was not possible to quantify. Meanwhile, the content of 3,4-DHB and BFCA did not seem to be affected by the nature of the cork powder, as it remained practically constant.

Compared to the results already described for the 30% ethanol extracts, the content of GA and *p*-HFL appears to decrease as the polarity of the extraction solvent decreases. The main difference between the aqueous and 30% ethanol extracts lies in the tannin richness, especially vescalagin found in the ethanol extracts [19].

The antioxidant properties of plant extracts are strongly linked to the presence of phenolic compounds and their structural characteristics. Usually, the number of hydroxy groups and conjugation and resonance effects improve the antioxidant activity [20]. Therefore, the enhanced antioxidant activity of extract prepared from P1 seems to be related to the abundance of phenolic compounds, especially tannins. However, other unidentified compounds may contribute to the improved antioxidant activity of this extract.

### 2.3. Effect of Cork-Derived Extracts on Cell Viability

**Figure 3:**
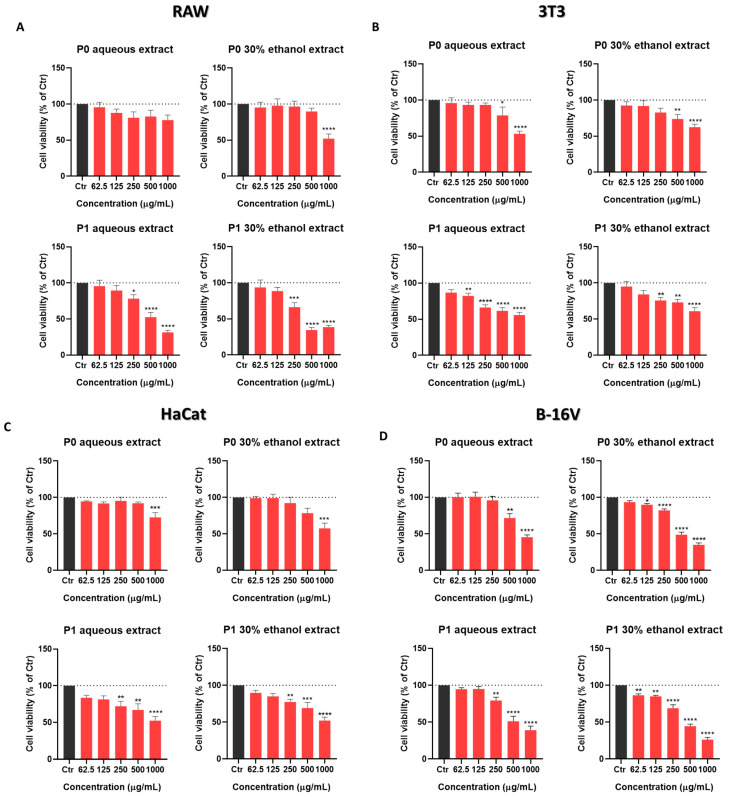
Cork extracts’ effect on cell viability. Macrophages (**A**), fibroblasts (**B**), keratinocytes (**C**), and melanocytes (**D**) were plated and exposed to different concentrations of cork extracts for 24 h. Resazurin assay was further performed to assess cell viability. Data correspond to mean ± SEM of at least three independent experiments and are represented as % of control cells (Ctr, black bars). Statistical analysis: one-way ANOVA with Dunnett’s multiple comparisons test; *p* < 0.05 was considered significant: * *p* < 0.05, ** *p* < 0.01, *** *p* < 0.001, **** *p* < 0.0001, compared to control (Ctr).

The safety and bioactivities of the newly obtained P0 and P1 aqueous extracts as well as 30% ethanol extracts, previously reported to be the most promising for skin application, were subsequently evaluated. Firstly, the viability of macrophages (RAW), fibroblasts (3T3), keratinocytes (HaCaT), and melanocytes (B-16V), exposed to different concentrations of the extracts, was assessed using a resazurin assay (Figure 3).

Considering the results for the four cell lines, we chose to proceed with the subsequent studies at concentrations of 62.5 µg/mL and 125 µg/mL, which had no effect (RAW, HaCaT) or minimal effects (P1 extracts on 3T3 and B-16V) on cell viability. In fact, these extracts present a IC_50_ > 200 μg/mL (Table 2), and are thus considered weakly cytotoxic, as stated in the international guidelines provided by the National Cancer Institute (NCI) of the U.S. [21].

### 2.4. Effect of Cork-Derived Extracts on Inflammatory and Antioxidant Parameters

#### 2.4.1. Nitrite Levels Production by Macrophages

Inflammation involves the activation of macrophages, which produce and release a large amount of pro-inflammatory mediators including interleukins, tumor necrosis factor alpha, nitric oxide, and reactive oxygen species. Since sustained inflammation often underlies skin afflictions, the potential anti-inflammatory effect of cork extracts was evaluated in macrophages exposed to the extracts 30 min before incubation with the inflammatory stimulus lipopolysaccharide (LPS), an outer-membrane component of Gram-negative bacteria that activates innate immune system cells through Toll-like receptor 4 (TLR4) binding.

**Figure 4:**
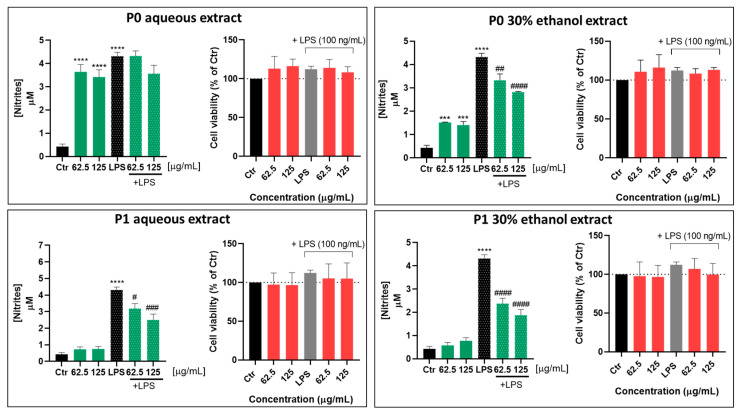
Cork extracts’ effect on macrophage NO production upon an inflammatory stimulus. Cells were plated and exposed to 62.5 and 125 µg/mL of cork extracts for 24 h, in the presence or absence of LPS (100 ng/mL). A Griess assay (green bars) and a resazurin assay (red bars) were performed to assess nitrite levels in the supernatant and cell viability (upon LPS exposure), respectively. The data correspond to the mean ± SEM of at least three independent experiments and are represented as nitrite concentration (in µM). Statistical analysis: one-way ANOVA with Tukey’s multiple comparison test; *p* < 0.05 was considered significant: *** *p* < 0.001 and **** *p* < 0.0001, compared to Ctr; # *p* < 0.05, ## *p* < 0.01, ### *p* < 0.001, and #### *p* < 0.0001, compared to LPS.

According to Figure 4, both extracts inhibited nitrite levels on macrophages induced by LPS, at both concentrations tested, being more pronounced at 125 µg/mL. Since the most promising results were obtained with the extracts at 125 µg/mL, we used this concentration in the next experiments.

#### 2.4.2. Expression of Inflammation- and Antioxidant-Related Genes

Since inflammation is tightly connected with oxidative stress, the effect of the cork extracts on inflammation- and antioxidant-related gene regulation was evaluated in macrophages and keratinocytes, respectively. A commercial cork ingredient by Hallstar Beauty containing a *Q. suber* extract was also tested. This compound was previously dissolved in culture medium to a concentration of 125 µg/mL, before being added to the cells

**Figure 5:**
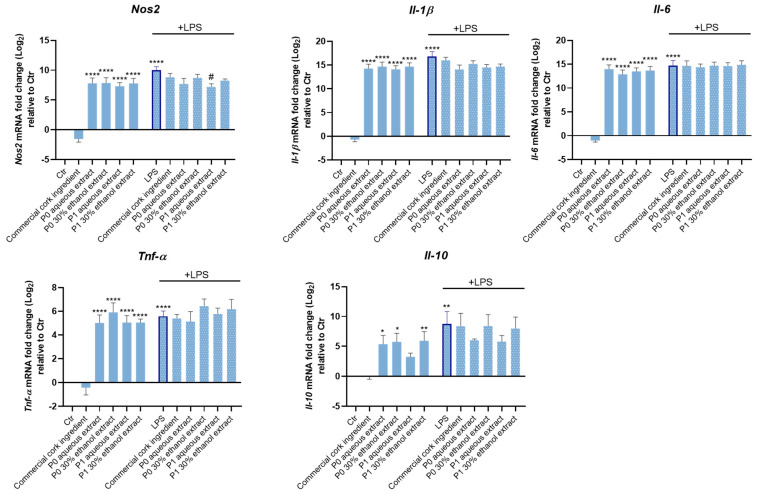
Cork extracts’ effect on inflammation-related gene expression. Macrophages were plated and exposed to 125 µg/mL of cork extracts, and to the commercial cork ingredient, in the presence or absence of LPS (100 ng/mL) for 6 h. The expression of the pro-inflammatory genes Nos2, Il-1β, Tnf-α, Il-6 and the anti-inflammatory gene Il-10 was determined by real-time RT-PCR. The data correspond to the mean ± SEM of at least three independent experiments and are expressed relative to the control cells (Log2 = 0). Statistical analysis: one-way ANOVA with Dunnett’s multiple comparison test; *p* < 0.05 was considered significant: * *p* < 0.05, ** *p* < 0.01, and **** *p* < 0.0001, compared to Ctr; # *p* < 0.05, compared to LPS.

According to the results, all the extracts increased the expression of the pro-inflammatory genes *Nos2* (which encodes the iNOS protein)*, Il-1β* (which encodes the Interleukin-1 beta (IL-1β) protein), *Tnf-α* (which encodes the Tumor Necrosis Factor-alpha (TNF-α) protein), *Il-6* (which encodes the IL-6 protein), and the anti-inflammatory gene *Il-10* (which encodes the IL-10 protein), in the absence of LPS (Figure 5). Interestingly, under an inflammatory milieu (i.e., cells exposed to LPS), P1 aqueous extract induced a significant reduction in *Nos2* gene expression, which was not observed with P0 extracts or the commercial cork ingredient, reinforcing its anti-inflammatory potential.

**Figure 6:**
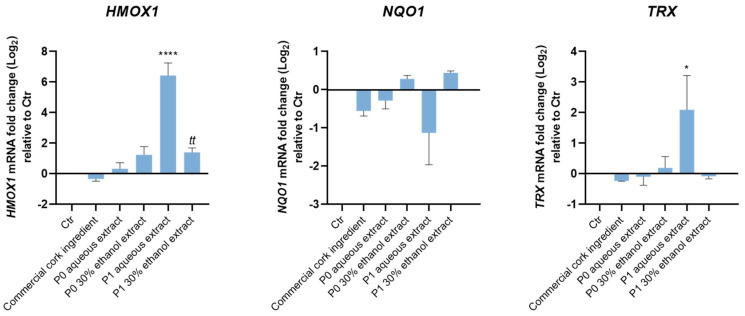
Cork extracts’ effect on antioxidant gene expression. Keratinocytes were plated and exposed to 125 µg/mL of cork extracts, and to the commercial cork ingredient, for 6 h. The expression of the antioxidant genes HMOX1, NQ01, and TRX was determined by real-time RT-PCR. The data correspond to the mean ± SEM of at least three independent experiments and are expressed relative to the control cells (Log2 = 0). Statistical analysis: one-way ANOVA with Dunnett’s multiple comparison test and unpaired *t*-test (t). *p* < 0.05 was considered significant: * *p* < 0.05, *tt p* < 0.01, and **** *p* < 0.0001, compared to Ctr.

To evaluate the potential antioxidant role of cork-derived extracts, the expression of the antioxidant genes *HMOX1* (which encodes the HMOX1 protein), *TRX* (which encodes the Thioredoxin (TRX) protein) and *NQ01* (which encodes the NAD (P)H dehydrogenase quinone 1 (NQ01) protein), all relevant cellular antioxidant enzymes, was addressed (Figure 6).

While both P1 extracts significantly increased the *HMOX1* gene expression, only P1 aqueous extract increased *TRX* gene expression. These results suggest an antioxidant role of P1 cork extracts that was not observed for P0 extracts or the commercial cork ingredient (Figure 6).

#### 2.4.3. Inflammatory and Antioxidant Protein Levels in Immune Cells

Since P1 extracts showed strong anti-inflammatory potential by significantly reducing nitrite levels and *Nos2* gene expression in macrophages exposed to LPS (P1 aqueous extract), and an antioxidant effect by significantly increasing *HMOX1* (both P1 extracts) and *TRX* (P1 aqueous extract) gene expression in keratinocytes, we selected P1 extracts to proceed with the subsequent experiments.

**Figure 7:**
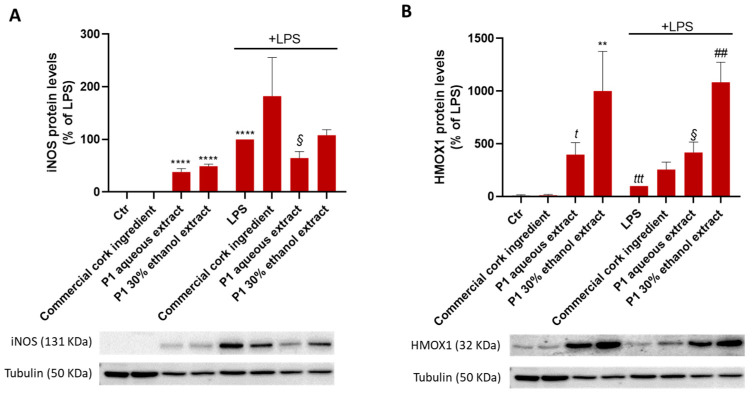
Cork extracts’ effect on the levels of the proteins iNOS and HMOX1 on immune cells. Macrophages were plated and exposed to 125 µg/mL of P1 cork extracts, and to the commercial cork ingredient, in the presence or absence of LPS (100 ng/mL) for 24 h. iNOS (**A**) and HMOX1 (**B**) protein levels were determined by Western blotting. Representative images of the blots are shown. The data correspond to the mean ± SEM of at least three independent experiments and are expressed as % of LPS. Statistical analysis: one-way ANOVA with Dunnett’s multiple comparison test (* and #) and unpaired *t*-test (*t* and *§*)*. p* < 0.05 is considered significant: ** *p* < 0.01 and **** *p* < 0.0001, compared to Ctr; *t p* < 0.05, and *ttt p* < 0.001, compared to Ctr; *§ p* < 0.05 and ## *p* < 0.01, compared to LPS.

Furthermore, the effect of cork extracts and the commercial cork ingredient on the levels of iNOS and HMOX1 proteins in macrophages exposed to LPS was evaluated (Figure 7).

Both P1 extracts increased the levels of iNOS per se, but only P1 aqueous extract significantly reduced iNOS protein levels induced by LPS (Figure 7A), in agreement with the results obtained for gene expression (Figure 5, *Nos2* graph). Furthermore, P1 extracts augmented the antioxidant enzyme HMOX1 in immune cells, in the absence or presence of an inflammatory stimulus (macrophages exposed to LPS) (Figure 7B). These results corroborate the capacity of P1 extracts in activating the antioxidant pathway in different cell types.

### 2.5. Effect of Cork-Derived Extracts on Skin Regeneration

**Figure 8:**
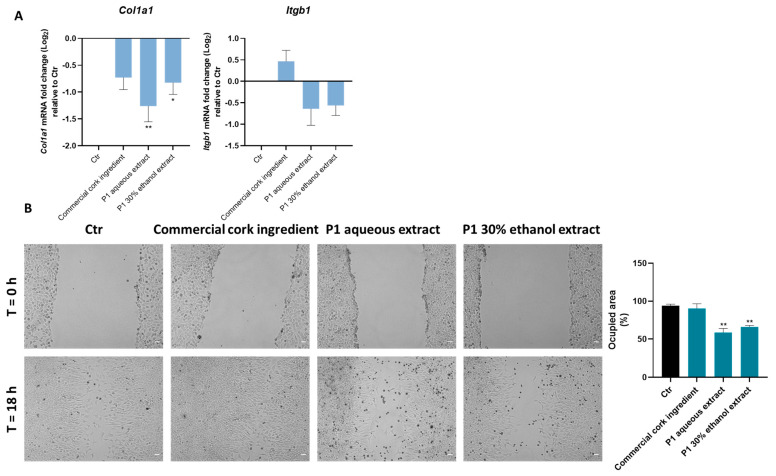
Cork extracts’ effect on skin regeneration. Fibroblasts were plated and exposed to 125 µg/mL of cork extracts, and to the commercial cork ingredient, for 6 h (**A**) or 18 h (**B**). (**A**) The expression of the genes that encode type I collagen (Col1a1) and Integrin beta-1 (Itgb1) proteins was determined by real-time RT-PCR. (**B**) A scratch wound assay was performed to evaluate the effect of cork extracts on cell migratory capacity and wound regeneration. The occupied area was calculated as explained in the Methods section, and the results are depicted in the graph. Representative microscopy images are shown in the panel (scale bar = 50 µm). The data correspond to the mean ± SEM of at least three independent experiments and are expressed relative to the control (Ctr). Statistical analysis: one-way ANOVA with Dunnett’s multiple comparison test. *p* < 0.05 was considered significant: * *p* < 0.05 and ** *p* < 0.01, compared to Ctr.

Skin cells, such as fibroblasts, are responsible for the maintenance of skin homeostasis and repair, and the loss of fibroblasts’ physiological activity is deeply involved in skin disorders and skin aging. Accordingly, the potential of P1 cork extracts in facilitating skin regeneration was explored in fibroblasts exposed to cork extracts and to the commercial cork ingredient. Thus, the expression of the genes *Col1a1* (which encodes for Type I collagen, essential for skin integrity) and *Itgb1* (which encodes for Integrin beta 1 protein, which mediates fibroblast adhesion to collagen type I) (Figure 8A), as well as cell migratory capacity (through the Scratch wound assay, as explained in the Methods section) (Figure 8B), were determined.

Regarding the results depicted in the graphs, the extracts significantly decreased *Col1a1* gene expression and induced a slight decrease in *Itgb1* gene expression (Figure 8A). In accordance, both extracts significantly reduced the migratory capacity of fibroblasts (Figure 8B), strongly indicating that these extracts impair cell migration and should be further explored in the context of anti-cancer therapies.

### 2.6. Anti-Senescent Effect of Cork-Derived Extracts

**Figure 9:**
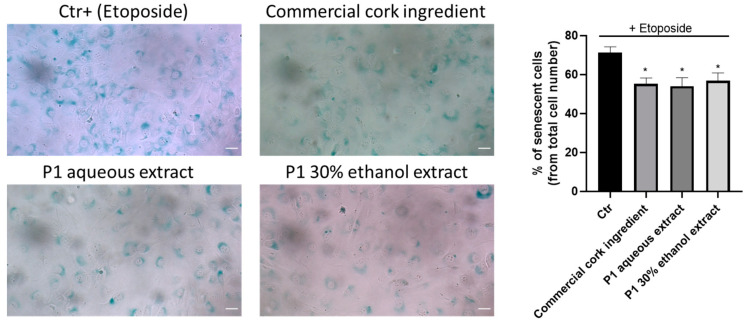
Anti-senescence effect of cork extracts. Fibroblasts were plated and exposed to etoposide (Ctr+; 12.5 µM), a senescence inducer, for 24 h and allowed to recover in the presence of 125 µg/mL of PC cork extracts, and of the commercial cork ingredient, for 72 h. Cell senescence was assessed using a commercially available beta-galactosidase staining kit. The number of senescent cells (stained with a distinct blue color resulting from beta-galactosidase activity) was visualized by microscopy. Representative microscopy images are shown (scale bar = 20 µm). The data correspond to the mean ± SEM of at least three independent experiments and are expressed relative to the control (Ctr). Statistical analysis: one-way ANOVA with Dunnett’s multiple comparison test. *p* < 0.05 was considered significant: * *p* < 0.05, compared to Ctr.

Since aging is associated with cell senescence, the anti-senescence capacity of the most promising extract, P1, was evaluated in etoposide-induced senescent fibroblasts, followed by exposure to P1 extracts, and to the commercial cork ingredient (Figure 9).

As we can observe in Figure 9, the extract, as well as the commercial cork ingredient, reduced the number of senescent cells, suggesting that these extracts possess anti-aging potential.

### 2.7. EpiSkin™ Skin Irritation Test

**Figure 10:**
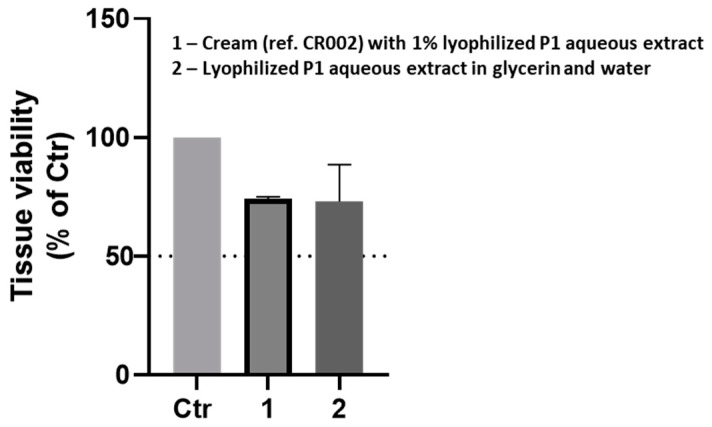
EpiSkin™ Skin Irritation Test. The EpiSkin™ human epidermis model (EpiSkin™) was used to assess the skin irritation potential of P1 aqueous extract, according to the OECD TEST 439 protocol. The compound tested is considered an irritant when the mean tissue viability is equal to or less than 50%.

Finally, we determined the irritation potential of P1 aqueous extract in vitro, using a commercial reconstructed human epidermis model (EpiSkin™), in agreement with the OECD Test 439 Guideline. Briefly, the tissues were exposed to the extract and extract-based formulation, and their viability was determined by an MTT assay (as explained in the Methods section). According to this skin irritation test, if the mean tissue viability is equal to or less than 50%, the tested chemical is considered an irritant, which was not the case when testing our samples (Figure 10). Thus, the P1 aqueous extract might be considered safe for human use.

### 2.8. Preformulation Studies with P1 Aqueous Extract

Cork powder is a naturally derived ingredient, and its color cannot be precisely controlled across different batches of extract. Therefore, color is a critical parameter for assessing batch variability and ensuring quality control, as it directly impacts the coloration of the final formulation in which the extract will be incorporated. For the P1 aqueous extract, the color was identified as brown, corroborated by colorimetric measurements: 50.39 ± 0.15 for the lightness parameter (L*), 5.71 ± 0.26 for the red/green chromatic coordinate (a*), and 15.55 ± 0.47 for the yellow/blue chromatic coordinate (b*). The pH of the P1 aqueous extract was 5.30 ± 0.01.

**Figure 11:**
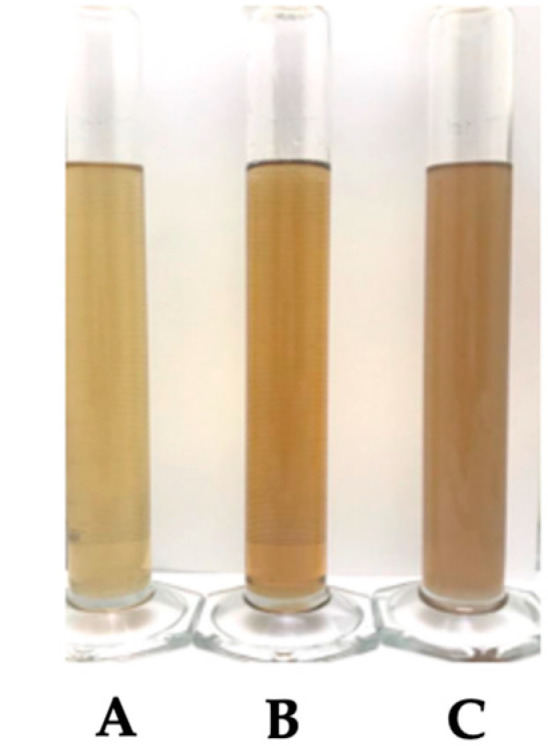
Solubility test of the P1 aqueous extract. (**A**): water; (**B**): glycerin; (**C**): 1,3 butyleneglycol.

The solubility of the P1 aqueous extract was evaluated to identify suitable solvents for incorporation into cosmetic formulations. The extract proved to be soluble in water and glycerin at a ratio of 1 g/L and insoluble in 1,3-butyleneglycol (Figure 11).

### 2.9. Formulation of a Cream with P1 Aqueous Extract

**Figure 12:**
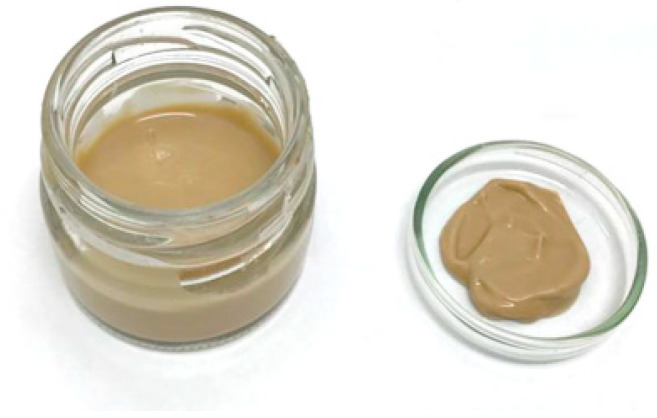
Appearance of the face cream formulation with 1% P1 aqueous extract.

The face cream formulation prepared with 1% P1 aqueous extract had a homogeneous appearance with a brownish color (Figure 12). This brown color was confirmed by the colorimetric measurements of 51.35 ± 0.00 for the L* parameter, 3.09 ± 0.00 for the a* chromatic coordinate, and 16.31 ± 0.01 for the b* chromatic coordinate. The pH of the formulation was 5.19 ± 0.01. The absence of phase separation in the centrifugation test showed that the formulation remained homogeneous, thus confirming its physical stability.

**Figure 13:**
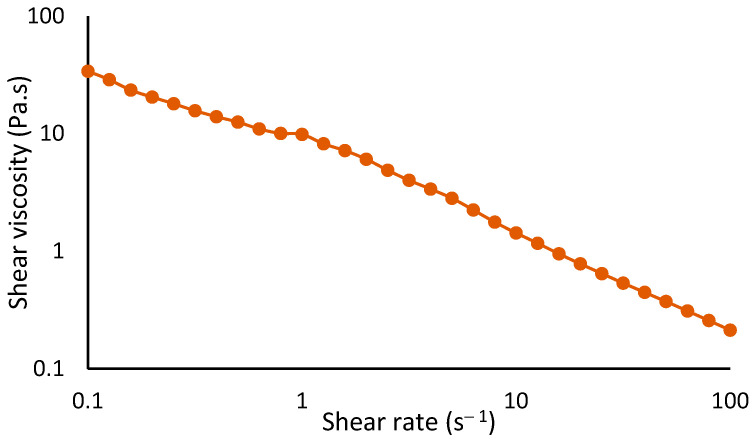
Flow curve obtained for formulation with P1 aqueous extract.

In terms of flow behavior, the formulation exhibited non-Newtonian shear-thinning properties, where shear viscosity decreased with increasing shear rate (Figure 13). The power law rheological model was fitted to the results with a consistency coefficient (K) value of 6.89 ± 2.40, a flow index (n) value of 0.20 ± 0.01, and an R^2^ value of 0.949 ± 0.025. The n value lower than 1 confirms that the formulation has shear-thinning behavior, characterized by higher viscosity at lower shear rates and a reduction in viscosity as the shear rate increases. This type of flow behavior is suitable for topical application.

## 3. Discussion

Aligned with the principles of the circular economy, which emphasize the reduction in waste, this study focused on adding value to two different cork powders, P0 and P1, widely found in the cork industry. Following the same idea as a previous work published by our group, aqueous extracts of P1 powder were first prepared to evaluate the influence of parameters such as temperature, time, and number of extraction cycles on the antioxidant activity. Once the extracts showed promising activity and to ensure the sustainability of the extraction process, it was established that the optimized conditions would involve only single extractions of 1 h at room temperature. Therefore, these optimized conditions were applied to both cork powders to prepare new extracts, using double the amount of water to improve the yield of extraction. Although these conditions allowed a slight increase in the yield (from 6% to 10%), the extraction method should be further optimized to allow greater improvement in the yield for large-scale preparation. The evaluation of the DPPH-scavenging effect of P0 and P1 aqueous extracts confirmed their potential antioxidant activity, with the P1 extract (IC_50_ = 7.5 ± 0.1 μg/mL) being more active than P0 (IC_50_ = 13.5 ± 0.8 μg/mL) (Figure 1B). These results were in accordance with those obtained in the previous study with the hydroalcoholic extracts, reinforcing the importance of the particle size of cork powder, as well as the possible different chemical compositions, in the potential antioxidant activity of the extracts [19].

The richness in phenolic compounds was evaluated by quantification analysis using a validated LC-UV method, which indicated the antioxidant potential of these extracts. It was observed that aqueous extracts had a higher number of tannins and phenolic acids. For P1 extract, castalagin was the major compound identified, while for P0 extract, it was *p*-HFL. Therefore, it is thought that the presence of tannins in the extracts may be beneficial for their antioxidant activity. However, there may be other unidentified compounds that contribute to the enhanced antioxidant activity of this extract.

Encouraged by the promising results of the 30% ethanol extracts, as well as the sustainable, safe, and non-toxic nature of water-based extracts, both extracts were chosen for further investigation into their potential skin-related activities. To accomplish this, the toxicity (assessed based on the impact on cell metabolism), anti-inflammatory, antioxidant, skin regenerative, and anti-senescence properties of the cork extracts were further evaluated through in vitro approaches. Accordingly, we used four cell lines representative of the skin, specifically of the epidermis (keratinocytes) and dermis (fibroblasts, melanocytes, and macrophages).

Cell viability alterations, upon exposure to cork-derived extracts derived from P0 and P1 powder, were determined, and the non-toxic concentrations were extrapolated. Indeed, several concentrations were tested in the four cell lines, and the concentrations of 62.5 µg/mL and 125 µg/mL, with no or minimal effect on cell viability, were selected for further evaluation. The anti-inflammatory and antioxidant capacity, two biological processes that are tightly connected, were studied. Inflammation is a central and intricate biological process, accomplished by the immune system in order to preserve normal tissue homeostasis, after injury or infection. Once triggered, it involves immune cell activation along with the overproduction and release of pro-inflammatory mediators by macrophages, including interleukins (e.g., IL-1, IL-6, IL-8), prostaglandins, tumor necrosis factor alpha (TNF-α), NO, and ROS. Prolonged inflammation results in detrimental health outcomes that might lead to a disease state, including skin conditions [22]. iNOS has a central role in immune activation and inflammation. This enzyme is induced in several types of cells in the presence of an inflammatory stimulus (e.g., LPS), producing large quantities of NO, an important signaling molecule in the organism, including in sustaining the skin and its milieu [23,24]. NO has been indicated as a potential antimicrobial agent to be applied in the treatment of skin disorders; however, its dysregulated production leads to the appearance of several dermatologic diseases [25].

Therefore, the effect of cork extracts on NO levels, inflammation-related gene expression, and iNOS protein levels was evaluated in macrophages exposed to LPS.

Both extracts inhibited NO production in macrophages exposed to LPS at both concentrations tested, but to a greater extent at 125 µg/mL (which was thus used in subsequent studies). Of note, only the P1 aqueous extract was able to reduce *Nos2* gene expression and iNOS protein levels under an inflammatory context (cells exposed to LPS). Surprisingly, these effects were not observed with the commercial cork ingredient that claims anti-inflammatory and antioxidant properties, which was simultaneously tested using the same experimental conditions.

Moreover, all extracts increased the expression of the pro-inflammatory genes *Nos2*, *Il-1β*, *Tnf-α*, and *Il-6* in the absence of LPS. However, this effect was not exacerbated in the presence of LPS. As observed by others [26], in the absence of an inflammatory stimulus, some compounds induced the release of pro-inflammatory mediators (such as TNF-α and IL-6). The authors suggested that by inducing a mild pro-inflammatory effect, these compounds activate the immune system to immediately fight infections [26]. Accordingly, the increase in NO production and in pro-inflammatory gene expression induced by both extracts alone might indicate antimicrobial potential, which should be further explored.

It is well established that elevated ROS levels induce oxidative stress, impairing the cellular oxidative defense system and significantly contributing to the onset of skin disorders, cellular aging, or senescence, and, consequently, to aging-related diseases [27]. Therefore, maintaining redox balance is a key issue in the development of new anti-aging products or new therapeutics for skin diseases. Macrophages, fibroblasts, and keratinocytes are involved in the maintenance of skin organ equilibrium, and are thus routinely used as in vitro models to study oxidative stress [28,29].

These results show that both extracts, but not the commercial cork ingredient, increased the expression of *HMOX1* and *TRX* (only P1 aqueous extract) genes in keratinocytes. These genes encode for HMOX1 and TRX proteins, respectively, two important antioxidant enzymes activated to mitigate excessive ROS. Of note, P1 aqueous extract, by inducing *TRX* gene expression, might exert a relevant antioxidant role in the skin. For more than three decades, thioredoxin reductases were shown to be associated with cell membranes and can reduce free radicals at the surface of the skin [30]. In fact, currently, thioredoxin is broadly used in skincare products as an antioxidant, mostly in conjunction with glutaredoxin and glutathione.

In addition, both extracts increased HMOX1 protein levels per se and under an inflammatory milieu (macrophages exposed to LPS), which corroborates the previous results and strongly suggests that P1 extracts might trigger cell antioxidant mechanisms.

Accordingly, the previous results support the anti-inflammatory and antioxidant activity of P1 extracts (compared to P0), which is relevant to the skin. Therefore, P1 extracts’ role in skin regeneration and cell senescence was further evaluated in fibroblasts. Fibroblasts, the major cell type in the dermis, are involved in skin injury recovery, and their activity loss seems to be a crucial factor for skin aging [31,32]. Hence, the expression of the genes encoding for Type I Collagen (*Col1a1*) and Integrin β1 (*Itgb1*) proteins, fibroblast migratory capacity, and etoposide-induced senescence in fibroblasts exposed to P1 extracts was investigated. Integrins are a family of cell-surface receptors that mediate attachment to the extracellular matrix. The family member integrin β1, which mediates fibroblast adhesion to collagen type I (essential for skin integrity), was shown to be required for cell survival and tissue repair in fibroblasts [33]. According to the results obtained, P1 extracts do not promote skin repair since both diminished the expression of *Col1a1* and *Itgb1* genes and, as expected, significantly reduced fibroblast migration (which was not observed with the commercial cork ingredient). In contrast, cork-derived extracts, as well as the commercial cork ingredient, reduced the number of senescent fibroblasts.

Regarding cosmetic application, the P1 aqueous extract was characterized by a brownish color, which imparted a similar coloration to the formulation in which it was incorporated. Although color may seem to limit consumer acceptance, nowadays, this organoleptic characteristic is more readily appreciated by consumers due to the perception of natural origin. The pH values of the extract and the formulation ranged from 4 to 6, indicating their skin compatibility for topical application. Furthermore, the shear-thinning behavior and physical stability exhibited under stress conditions by the formulation containing the P1 aqueous extract highlight its applicability as cosmetic ingredient.

To conclude, these findings indicate that P1 cork-derived extracts, especially aqueous extract, possess significant anti-inflammatory and antioxidant properties, more prominent than the commercial cork ingredient, and were shown to exert an anti-senescence effect. In addition, since this extract is not a skin irritant (as evaluated by the EpiSkin^TM^ Irritation Test), it is well suited for incorporation into cosmetic products, underscoring its potential as a bioactive ingredient for skincare applications. Further investigation is warranted to elucidate their mechanisms of action and maximize their efficacy in this context. To substantiate efficacy claims, in vivo studies conducted with the final cosmetic formulations could also be carried out.

## 4. Material and Methods

### 4.1. Cork-Derived Powder and Extracts

Dimas and Silva collected the ground cork powders at two stages of the industrial cycle. Cork planks were extracted from the tree, boiled, and ground, producing 3 to 30 mm pieces. P0 powder (0–2 mm grain size) is the outer part released in this process. After this step, the 3 to 30 mm pieces were dried with hot air and went through another grinding step to obtain a 0.5–9 mm size. At this point, the cork powder was sieved with 180 µm and 63 µm mesh sieves and the fraction above 63 µm was collected to obtain the cork powder P1.

Aqueous and 30% ethanol extracts of both P0 and P1 were obtained following the procedure described in previous work published by our group [19,34]. In brief, 2.5 g of P0 or P1 was stirred into 100 mL of water/30% ethanol at room temperature using a magnetic multistirrer (Velp Scientifica, Usmate Velate, MB, Italy). At the end of extraction, the mixtures were filtered through a Büchner funnel with a fiberglass filter membrane (1.20 μm pore size), and the solvent was removed under reduced pressure in a Büchi Rotavapor R-210 (Büchi, Flawil, Switzerland). The resulting extracts were freeze-dried (Telstar, Terrassa, Spain).

The cork powder extracts were diluted in culture medium to a final concentration of 1 mg/mL.

### 4.2. Determination of Antioxidant Activity by DPPH

The DPPH radical scavenging activity was assessed using a UV/visible spectrophotometer following the method established by Brand-Williams et al. [35]. On a 96-well microplate, 100 μL of a freshly prepared hydroalcoholic 150 μM (ethanol 96°) DPPH solution was mixed with 100 μL of extract solutions (ethanol 96°) at different concentrations (from 0.78 to 50 μg/mL). The reaction was monitored in a BioTek Synergy^TM^ HT microplate reader (Bio-Tek Instruments, Inc., Winooski, VT, USA) after 30 min at 517 nm, and the results were compared to the initial absorbance of the DPPH solution. The assay was performed in quadruplicate and repeated at least three times. The percentage of inhibition of DPPH radical activity was calculated using Formula (1):

(1) $$\mathrm{Inhibition}\;\mathrm{of}\;\mathrm{DPPH}\;\mathrm{radical}\;(\%)=\left(1-\frac{{Abs}_{problem}-{Abs}_{problem\;blank}}{{Abs}_{control}-{Abs}_{reaction\;blank}}\right)\times 100$$

The antioxidant activity, expressed in μg/mL, was measured based on the concentration required to reduce the initial DPPH radical absorption by 50% (IC_50_).

### 4.3. Analysis Using LC

The preliminary analysis was performed in a Luna 3 μm PFP (2) column from Phenomenex (Torrance, CA, USA) using (A) H_2_O:ethanol:formic acid (93.5:5.5:1, *v:v:v*) and (B) ethanol:formic acid (99:1, *v:v*) and mobile phase in a linear gradient (0–10 min, 100% A; 10–40 min, 100–0% A; 40–50 min, 100% A), followed by re-equilibration of the column for 10 min. The flow rate was set to 0.5 mL/min, the column temperature to 30 °C, with an injection volume of 20 μL, and the wavelength detection to both 280 nm and 380 nm. Samples were dissolved in a mixture of 50:50 (*v:v*) H_2_O:EtOH to a final concentration of 1 mg/mL and filtered with a 0.2 μm hydrophilic PTFE syringe filter prior to injection. The LC system constituted a Shimadzu LC-20AD pump, a Shimadzu DGV-20A5 degasser, a Rheodyne 7725i injector, an SPD-M20A diode array detector (DAD), and Shimadzu LC Lab Solutions software, version 3.50 SP2 (Kyoto, Japan).

The identity of the target compounds was confirmed by LC-HRMS performed at CEMUP—Centro de Materiais da Universidade do Porto, using the previously reported conditions [19].

The LC-UV method was validated according to the International Conference on Harmonization (ICH) guidelines for parameters of linearity, limits of detection (LOD) and quantification (LOQ), accuracy and precision [36], on an Ultra-High-Performance Liquid Chromatography (UHPLC) Dionex UltiMate 3000 system equipped with a 3000 Quaternary Pump, a 3000 Variable Wavelength Detector set at 280 nm, and Chromeleon^TM^ 7.0 software for data processing (Thermo Fisher Scientific, Bremen, Germany).

The calibration curves demonstrated excellent linearity, with correlation coefficients exceeding 0.99. The limits of detection (LOD) ranged from 0.04 to 8 μg/mL, while the limits of quantification (LOQ) varied between 0.1 and 25 μg/mL. The accuracy was between 80% and 117%, which falls within the acceptable range for quantitative analysis in complex matrices. The precision, measured as the relative standard deviation (RSD%), was below 20% for all compounds tested.

### 4.4. Cell Culture

The mouse leukemic monocyte macrophage cell line (RAW 264.7—ATCC number TIB-71) was cultured in DMEM medium (pH 7.2) supplemented with fetal bovine serum (FBS, 10%) (both from Life technologies, Carlsbad, CA, USA), glucose (up to 25 mM), sodium bicarbonate (17.95 mM), penicillin (100 U/mL), and streptomycin (100 µg/mL) (all from Sigma-Aldrich, St. Louis, MO, USA).

Mouse skin fibroblasts (3T3, obtained from ATCC CRL-1658) and the human keratinocyte cell line (HaCaT, CLS 300493, acquired from DKFZ) were grown in DMEM medium (pH 7.2) supplemented with heat-inactivated FBS (10%) (both from Life technologies, Carlsbad, CA, USA), glucose (up to 25 mM), sodium bicarbonate (35.9 mM), penicillin (100 U/mL), and streptomycin (100 µg/mL) (all from Sigma-Aldrich, St. Louis, MO, USA).

Mouse melanocytes established from a B16 melanoma tumor (B16V cells; DSMZ ACC-370, Braunschweig, Germany) were cultured in DMEM medium (pH 7.2) containing glucose (5.6 mM) and sodium bicarbonate (44 mM), supplemented with heat-inactivated FBS (10%), 50 U/mL penicillin, and 50 μg/mL streptomycin (all from PAN-Biotech GmbH, Aidenbach, Germany).

The cells were maintained at 37 °C in a humidified atmosphere of 95% air and 5% CO_2_. Before reaching confluence, the cells were detached with a cell scraper (RAW 264.7), or with trypsin (3T3, HaCaT and B-16V) and further subcultured in fresh culture media.

### 4.5. Metabolic Capacity (Resazurin Assay)

The murine RAW 264.7 macrophages (50,000 cells/well), murine 3T3 fibroblasts (10,000 cells/well), human HaCaT keratinocytes (20,000 cells/well), and murine B-16V melanocytes (30,000 cells/well) were plated in a 96-well plate with a final volume of 0.2 mL/well, for 24 h (RAW 264.7, 3T3, and HaCaT) or 48h (B-16V). The next day, the medium was completely removed and replaced with fresh culture medium with cork-derived extracts. Different concentrations of the extracts (in µg/mL) were tested for different time periods, as depicted in the graphs.

After 24 h of incubation, cell viability was indirectly assessed using a resazurin reduction assay, as described elsewhere [37]. Absorbance (RAW 264.7, 3T3, and HaCaT) and fluorescence (B-16V) were read at excitation/emission wavelengths of 570/620 nm and 530/590 nm, respectively, with a Synergy^TM^ HT multi-mode microplate reader (BioTek, Bad Friedrichshall, Germany). Metabolically active cells reduce resazurin (a non-fluorescent blue dye) into resorufin (pink colored and fluorescent form) and, hence, their number correlates with the magnitude of dye reduction. The tests in all conditions were performed in duplicate and the results are expressed as a percentage of the control.

IC_50_ (half-maximal inhibitory concentration) was calculated by plotting the log concentration values of the extracts against the percentage of viable cells.

### 4.6. Determination of Nitric Oxide (NO) Production (Griess Assay)

The murine RAW 264.7 macrophages (50,000 cells/well) were plated in duplicate in a 96-well plate with a final volume of 0.2 mL/well, and simultaneously exposed to cork extracts (62.5 and 125 µg/mL) and to the pro-inflammatory stimuli lipopolysaccharide (LPS, 100 ng/mL, 1 h after extract delivery) for 24h. NO production was further determined through a colorimetric Griess assay [38], which measures nitrite accumulation in the culture supernatants. Briefly, equal volumes of cell culture supernatants and Griess reagent [1% (*w*/*v*) sulphanilamide in 5% (*w*/*v*) phosphoric acid and 0.1% (*w*/*v*) N-(1-naphthyl)-ethylenediamine dihydrochloride] were mixed and incubated at room temperature (RT) for 30 min. The absorbance was measured at 550 nm in a Synergy^TM^ HT multi-mode microplate reader (BioTek, Bad Friedrichshall, Germany). Nitrite concentration was calculated through a regression analysis of a sodium nitrite standard curve.

### 4.7. Gene Expression (Real Time RT-PCR)

The murine RAW 264.7 macrophages (1 × 10^6^ cells/well), murine 3T3 fibroblasts (290,000 cells/well), and human HaCaT keratinocytes (500,000 cells/well) were plated in a 6-well plate with a final volume of 2 mL/well and incubated with the extracts (125 µg/mL) (and 100 ng/mL LPS, added 1h after cork extracts, in inflammation studies) for 6 h.

Total RNA was further extracted with NZYol reagent, according to the manufacturer’s instructions, and its concentration was determined through OD260 measurement using a NanoDrop spectrophotometer (Thermo Scientific, Wilmington, DE, USA). Samples were stored in RNA Storage Solution at −80 °C until use. An amount of 2 µg of RNA was transcribed to cDNA with an NZY First-Strand cDNA Synthesis kit (NZYtech) on a C1000^TM^ Thermal Cycler (Bio-Rad, Hercules, CA, USA), and each sample product was amplified in duplicate with an NZYSpeedy qPCR Green Master Mix (2x) kit via real-time reverse transcriptase-polymerase chain reaction (RT-PCR) on a CFX Connect^TM^ Real-Time System (Bio-Rad). After amplification, a threshold for each gene and Ct (Cycle threshold) was calculated and normalized using *Hprt-1* (Hypoxanthine-guanine phosphoribosyl transferase) as a reference gene. Mouse primers (Table 3) were obtained from Sigma (except mouse *Hprt1*); mouse *Hprt1* (Table 3) and human primers (Table 4) were designed with Beacon Designer software version 7.7 (Premier Biosoft International, Palo Alto, CA, USA) and obtained from NZYtech. The results were analyzed using Bio-Rad CFX Maestro^TM^ 1.1 system software (Hercules, CA, USA).

Since the results are presented as ratios of chemical-treated samples/untreated (control) cells, a two-base logarithmic transformation was used to make the observations symmetric and closer to a normal distribution. If x represents the gene fold change in one sample, then the two-base logarithmic transformation (log_2_ (x)) is ln (x)/ln (2). Hence, fold changes of 2 and 0.5 correspond to mean log_2_ values of 1 and −1, respectively.

### 4.8. Protein Levels (Cell Extracts and Western-Blotting)

The murine RAW 264.7 macrophages (1 × 10^6^ cells/well) were plated in a 6-well plate with a final volume of 2 mL/well for 24 h. After this, the cells were incubated with cork extracts (125 µg/mL) (and 100 ng/mL LPS, added 1h after cork extracts in inflammation studies) for another 24 h.

The cells were then collected, centrifuged at 500× *g* for 5 min at 4 °C, and washed in ice-cold PBS, pH 7.4 (2×). The pellet was further incubated in 100 µL of RIPA lysis buffer [50 mM Tris–HCl (pH 8.0), 1% (*v*/*v*) Nonidet P-40, 150 mM NaCl, 0.5% (*w*/*v*) sodium deoxycholate, 0.1% (*w*/*v*) SDS, 2 mM EDTA], freshly supplemented with 1 mM dithiothreitol, and protease (1:7) and phosphatase (1:10) inhibitor cocktails for 30 min in ice. The nuclei and the insoluble cell debris were removed by centrifugation at 4 °C, at 12,000× *g* for 10 min. The supernatant fraction with the cell extracts was collected and stored at −80 °C until use. The protein concentration was determined using the bicinchoninic acid method, and the cell lysates were denatured in 4× concentrated loading buffer [0.25 M Tris pH 6.8, 4% (*w*/*v*) SDS, 200 mM DTT, 20% (*v*/*v*) glycerol and bromophenol blue] at 95 °C, for 5 min.

Briefly, 30 µg of protein was electrophoretically separated on a 10% (*v*/*v*) sodium dodecyl sulfate–polyacrylamide gels (SDS-PAGE) at 130 V and transferred to a PVDF membrane. The membranes were blocked with 5% (*w*/*v*) non-fat dry milk in Tris-buffered saline [(TBS): 150 mM NaCl, 25 mM Tris-HCl, pH 7.6] containing 0.1% (*v*/*v*) Tween 20 (TBS-T) for 1 h at RT. The membranes were then incubated overnight at 4 °C with the primary antibodies against iNOS (1:1000) or HMOX1 (1:500), and further washed for 30 min with TBS-T. After incubation with anti-mouse horseradish peroxidase conjugated secondary antibody (1:10,000), for 1 h at RT, the membranes were washed with TBS-T (3×) and the blots visualized by chemiluminescence using ImageQuant^TM^ LAS 500 (GE Healthcare, Chicago, IL, USA). The generated signals were analyzed using total lab TL120 software. β-tubulin protein (1:20,000) was used as loading control.

### 4.9. Scratch Wound Assay

Cell migration was evaluated USING a scratch wound assay as follows. The murine 3T3 fibroblasts (250,000 cells/well) were plated in a 12-well plate with a final volume of 1 mL/well for 24 h. After reaching approximately 95% confluence, a wound was made via a perpendicular scratch with a P10 pipette tip in the cell layer, and eight photographs/condition were taken (T0 = 0 h) under a Zeiss Axio HXP IRE 2 microscope with an EC plan-Neofluar 10× objective. Next, the cells were exposed to cell culture medium, alone (control) or with cork extracts (125 µg/mL), with 2% FBS (*v*/*v*) to reduce the proliferation rate during the experiment. After 18 h (T18), images at the same coordinates as in T0 were recorded. The cell-free areas at T0 and T18 were measured using ImageJ software (version ImageJ 1.53c). The migration rate was then calculated using the formula T0_cell free area_—T18_cell free area_ = occupied area. The data are expressed as % of occupied area relative to T0 ((occupied area/T0_cell free area_) × 100).

### 4.10. Cell Senescence (B-Gal Staining Kit)

Cell senescence was assessed using a commercially available beta-galactosidase staining kit (Cell Signaling Technology) according to the manufacturer’s protocol. Briefly, murine 3T3 fibroblasts (25,000 cells/well) were plated in a 12-well plate with a final volume of 1 mL/well for 24 h. After this, senescence was induced by incubating cells with 12.5 μM of etoposide for another 24 h. The etoposide was then removed, and the cells were washed with PBS. Then, the cells were allowed to recover in cell culture media with or without (control) cork extracts (125 µg/mL). After 72 h, the cells were fixed for 15 min using 1× fixative solution (provided in the commercial kit), followed by PBS washes, and incubated for 18 h with beta-galactosidase staining solution in a dry incubator at 37 °C without a CO_2_ supply. Different fields were viewed under a microscope (Zeiss Axio HXP IRE 2 microscope with an EC plan-Neofluar 10× objective) for blue color development and were photographed for image analysis (5–8 images per condition). Distinct blue color staining was indicative of beta-galactosidase activity. A quantitative analysis was carried out using ImageJ, and the percentage of senescent cells to total cells was calculated (mean ± SEM).

### 4.11. EpiSkin™ Skin Irritation Test

The EpiSkin™ human epidermis model (Episkin™) was used to assess the skin irritation potential of the selected cork-derived extract, according to the OECD TEST 439 guideline. The P1 aqueous extract was either tested in hydroglyceric solution (3.5 wt%) or incorporated into a cream (1 wt%). Briefly, the Episkin™ model (aged 13 days, small/0.38 cm^2^ surface area) was provided by Episkin™ (Lyon, France). Upon receipt, the culture inserts were removed from the agarose–nutrient solution and transferred into 6-well plates containing 2 mL/well of maintenance medium (provided by the manufacturer). The tissues were then incubated for 24 h at 37 °C in a humidified atmosphere of 5% CO_2_ before use.

The next day, the Episkin™ tissues (3 tissues/PBS and 2 tissues/sample) were incubated with PBS (negative control, ngC = 100% viability) or the formulations (10 mg [26 µg/cm^2^]) for 15 min at room temperature and further washed with PBS (1 mL, 25 times = 25 mL PBS). Then, the tissues were placed in new 12-well plates containing 0.3 mg/mL MTT (Thiazolyl Blue Tetrazolium Bromide) solution, in maintenance medium (final volume per well = 1 mL), and incubated for an additional 3 h at 37 °C in a humidified atmosphere of 5% CO_2_. After, the tissues were immersed in acidified isopropanol (0.04 N HCl in isopropanol) for 18 h at 4 °C to extract the formazan. At the end of the incubation time, 200 µL of each sample was transferred to a 96-well plate (in duplicates) and the absorbance read at 570 nm (A570).

### 4.12. Preformulation Studies with P1 Aqueous Extract

P1 aqueous extract was considered the most promising due to its high bioactivity and contribution to more sustainable processes and was further evaluated for cosmetic applicability. The color and pH of the P1 aqueous extract were assessed following the methods outlined by [34]. The solubility of the P1 aqueous extract was evaluated following the OPPTS 830.7840 guidelines [39]. Water, glycerin, and 1,3-butyleneglycol were tested as solvents.

### 4.13. Formulation of a Cream with P1 Aqueous Extract

A face cream formulation was prepared for the incorporation of 1% P1 aqueous extract (Table 5).

Phases A and B were heated to a temperature range of 65–70 °C, combined, and homogenized using a high-performance homogenizer (IKA^®^ T25 digital ULTRA TURRAX^®^, IKA Werke GmbH & Co. KG, Staufen im Breisgau, Germany) under continuous stirring at 10,000 rpm for 15 min. Subsequently, the mixture was stirred at a reduced speed with a mechanical stirrer (Heidolph RZR 2041, Heidolph Instruments GmbH & Co. KG, Schwabach, Germany) until the temperature decreased to 40 °C. At this point, phase C was added and also subjected to homogenization with a mechanical stirrer for an additional 10 min. A preliminary assessment of physical stability was conducted using a centrifugation test, performed at 3500 rpm for 30 min.

The color and pH of the face cream formulation were evaluated following the methodologies previously described. Measurements were performed in triplicate. Additionally, the rheological properties of the formulation were studied. The flow behavior was evaluated on a Kinexus Prime lab+ Rheometer (Malvern Panalytical, Malvern, UK) at 25 °C. A cone-and-plate geometry was employed, featuring a cone angle of 4°, a diameter of 40 mm, and a working gap of 0.150 mm. Measurements were conducted over a shear rate range of 0.1 s^−1^ to 100 s^−1^. The data were analyzed by fitting the power law rheological model to the experimental results.

### 4.14. Assay Controls

Since the cream developed color when in contact with water (10 mg mixed with 90 µL of distilled water for 30 min), and both reacted with the MTT solution (10 mg mixed with 0.3 mg/mL of MTT solution for 3 h) additional controls were performed. Accordingly, one tissue was incubated with the cream and not exposed to MTT for non-specific color assessment (NSCliving), and one killed tissue (immersed in distilled sterile water overnight) was incubated with both samples for a non-specific reduction of MTT assessment (NSMTT). Calculations of tissue viability were made according to the following formulas:

Cream and extract 2 (i): % of tissue viability (i) = Abs570 (i)/Abs570ngC

Cream: Final %viability (i) = %tissue viability (i) − %NSMTT (i)

Extract: Final %viability (i) = %tissue viability (i) − NSCliving (i) − %NSMTT (i)

### 4.15. Statistical Analysis

The statistical analysis was performed using GraphPad Prism version 8.0 for Windows (GraphPad Software, San Diego, CA, USA; www.graphpad.com, accessed on 30 January 2019). For each experimental condition, the results are presented as the mean value ± SEM of at least three independent experiments. Comparisons between two groups were made using the two-tailed unpaired Student *t*-test, and multiple-group comparisons via one-way ANOVA analysis, with Dunnett’s or Tukey’s multiple comparison post-test. A value of *p* < 0.05 was considered significant (* *p* < 0.05, ** *p* < 0.01, *** *p* < 0.001, and **** *p* < 0.0001).

## Figures and Tables

**Table 1:** Quantification of compounds identified in P0 and P1 aqueous cork powder extracts.

Family	Compound	Concentration (µg/mg Dry Extract)	
		H_2_O	
		P0	P1
Tannins	Vescalagin	-	<LOQ
	Castalagin	10.4	28.2
Phenolic acids	GA	15.7	7.2
	3,4-DHBA	3.8	2.9
	*p*-HFL	44.9	<LOQ
	BFCA	1.3	1.3
	EA	7.8	12.8
Phenolic aldehydes	3,4-DHB	0.5	0.3
	4H3MCA	<LOQ	<LOQ
Coumarins	6,7-DHC	2.0	1.4

**Table 2:** IC_50_ of cork extracts.

	IC_50_ (µg/mL)			
	RAW	3T3	HaCat	B-16V
P0 aqueous extract	2728	1508	3149	1280
P0 30% ethanol extract	1888	1476	1688	651.5
P1 aqueous extract	661.2	872.5	867.5	712.5
P1 30% ethanol extract	497.8	1263	996.7	455.4

**Table 3:** Mouse primers.

Gene	Forward Primer	Reverse Primer
*Hprt-1*	5′ GTTGAAGATATAATTGACACTG 3′	5′ GGCATATCCAACAACAAAC 3′
*Nos2*	5′ CATCAACCAGTATTATGGCTC 3′	5′ TTTCCTTTGTTACAGCTTCC 3′
*Il-1β*	5′ GGATGATGATGATAACCTGC 3′	5′ CATGGAGAATATCACTTGTTGG 3′
*Tnf-α*	5′ GGATGAGAAGTTCCCAAATG 3′	5′ TGAGAAGATGATCTGAGTGTG 3′
*Il-6*	5′ AAGAAATGATGGATGCTACC 3′	5′ GAGTTTCTGTATCTCTCTGAAG 3′
*Il-10*	5′ CAGGACTTTAAGGGTTACTTG 3′	5’ ATTTTCACAGGGGAGAAATC 3’

**Table 4:** Human primers.

Gene	Forward Primer	Reverse Primer
*HPRT-1*	5′ TGACACTGGCAAAACAATG 3′	5′ GGCTTATATCCAACACTTCG 3′
*HMOX1*	5′ CCTGAGTTTCAAGTATCC 3′	5′ AACAACAGAACACAACAA 3′
*NQ01*	5′ GAGTCTGTTCTGGCTTAT 3′	5′ AACTGGAATATCACAAGGT 3′
*TRX*	5′ GCCGCTCGTCAGACTCCAG 3′	5′ GCAGCGTCCAAGGCTTCC 3′

**Table 5:** Composition of the face cream formulation with 1% P1 aqueous extract.

Phase	Ingredients	INCI Name	Amount (%)
A	Olivatis^®^ 18	Olive Oil Polyglyceryl-6 Esters (and) Sodium Stearoyl Lactylate (and) Cetearyl Alcohol	5.00
	Miglyol^®^ 812^®^	Caprylic/Capric Triglyceride	15.00
	Lanette O^®^	Cetearyl Alcohol	2.00
B	Xanthan gum	Xanthan gum	0.15
	Propylene glycol	Propylene glycol	2.00
	Water	Aqua	qs 100
C	P1 aqueous extract	-	1.00
	euxyl™ k 940	Phenoxyethanol (and) Benzyl Alcohol (and) Ethylhexylglycerin (and) Tocopherol	1.00

## Data Availability

The original contributions presented in the study are included in the article/supplementary material, further inquiries can be directed to the corresponding authors.

## Supplementary Materials

The following supporting information can be downloaded at: https://www.mdpi.com/article/10.3390/ph18010121/s1, Table S1: LC-HRMS data of identified compounds in the P0 H_2_O extract; Table S2: LC-HRMS data of identified compounds in the P1 H_2_O extract.
