# Supplementary material for: Sustainable Skincare Innovation: Cork Powder Extracts as Active Ingredients for Skin Aging
Source: Pharmaceuticals (Basel). 2025 Jan 17;18(1):121. doi: 10.3390/ph18010121 (PMC11769245; doi:10.3390/ph18010121)
Supplement: Supplementary file 1 [file pharmaceuticals-18-00121-s001.zip › pharmaceuticals-3408157-supplementary.pdf]

Table S1: LC-HRMS data of identified compounds in the P0 H_2O extract.

Compound	ESI/MSn	
	[M–H]-	MS²
GA	169.01422	125.02436
Castalagin	933.06342	-
3,4-DHBA	153.01930	109.02942
p-HFL	180.97289	-
3,4-DHB	137.02426	92.92008/109.02972
BFCA	291.01575	202.07878/247.02466
6,7-DHC	177.01933	133.02953
EA	300.99881	202.07893
4H3MCA	177.05591	-

Gallic acid: GA; Protocatechuic acid: 3,4-DHBA; Latifolicinin C acid: *p*-HFL; Protocatechuic aldehyde: 3,4-DHB; Brevifolincarboxylic acid: BFCA; Aesculetin: 6,7-DHC; Ellagic acid: EA.

Table S2: LC-HRMS data of identified compounds in the P1 H_2O extract.

Compound	ESI/MSn	
	[M–H]-	MS²
Vescalagin	933.06329	-
GA	169.01433	125.02441
Castalagin	933.06348	202.07887/300.99890/425.01431/467.02548/569.05786
3,4-DHBA	153.01933	109.02943
p-HFL	180.97301	-
3,4-DHB	137.02429	92.91986/109.02949
BFCA	291.01572	202.07878/247.02460
6,7-DHC	177.01927	105.03470/133.02956
EA	300.99887	202.07893
4H3MCA	177.05583	-

Gallic acid: GA; Protocatechuic acid: 3,4-DHBA; Latifolicinin C acid: *p*-HFL; Protocatechuic aldehyde: 3,4-DHB; Brevifolincarboxylic acid: BFCA; Aesculetin: 6,7-DHC; Ellagic acid: EA.
